# A green TLC densitometric method for the simultaneous detection and quantification of naphazoline HCl, pheniramine maleate along with three official impurities

**DOI:** 10.1186/s13065-022-00819-9

**Published:** 2022-04-04

**Authors:** Khadiga M. Kelani, Maha A. Hegazy, Amal M. Hassan, Mahmoud A. Tantawy

**Affiliations:** 1 Analytical Chemistry Department, Faculty of Pharmacy, Cairo University, Kasr el Aini Street, Cairo, 11562 Egypt; 2 Analytical Chemistry Department, Faculty of Pharmacy, Modern University for Technology and Information, El-hadaba El-Wosta, Mokatam, 5th district, Cairo, Egypt; 3 Chemistry Department, Faculty of Pharmacy, October 6 University, 6 October City, Giza Egypt

**Keywords:** Naphazoline, Pheniramine, Impurities, TLC, EHS tool

## Abstract

Impurity profiling of a pharmaceutical compound is now taking great attention during quality assessment of pharmaceuticals, as presence of small amount of impurities may affect safety and efficacy. In this work, a novel TLC chromatographic method coupled with densitometric detection was established for the simultaneous quantification of naphazoline HCl, pheniramine maleate and three of their official impurities, namely; naphazoline impurity B, pheniramine impurities; A & B. Chromatographic separation was carried out on TLC aluminum silica plates F254, as a stationary phase, using methanol: ethyl acetate: 33.0% ammonia (2.0: 8.0: 1.0, by volume), as a mobile phase. Plates were examined at 260.0 nm and International Council for Harmonisation (ICH) guidelines were followed for method’s validation. Important factors, such as; composition of mobile phase and detection wavelengths were optimized. Linearity was achieved over the ranges of 2.0–50.0 µg band^−1^ for naphazoline, 10.0–110.0 µg band^−1^ for pheniramine, 0.1–10.0 µg band^−1^ for naphazoline impurity B and 2.0–50.0 µg band^−1^ for both pheniramine impurities. The proposed method was assessed in terms of accuracy, precision and robustness where satisfactory results (recovery % ≈ 100% and RSD < 2) were obtained. The method was also applied for the simultaneous determination of naphazoline HCl and pheniramine maleate, in Naphcon-A^®^ eye drops, with respective recoveries of 101.36% and 100.94%. Method greenness was evaluated and compared to the reported HPLC one via environmental, health and safety tool. The developed method has much potential over the reported one of being simple, selective, economic and time saving for the analysis of the five cited compounds.

## Introduction

A great attention was given, by modern pharmaceutical analysis, to impurity profiling of the drug substances, as the presence of impurities, even in trace amounts, may affect the quality, potency and safety of the drug product [1]. In a different manner, Thin Layer Chromatography (TLC) is one of the most familiar and adaptable techniques used in detection and quantification of related impurities in the pharmaceutical filed. It has several advantages including; simplicity, cost-effectiveness, rapidness as well as good resolving power with accurate quantification of multicomponent mixtures [2].

Naphazoline HCl (NPZ) is chemically known as 2-(naphthalen-1-ylmethyl)-4, 5-dihydro-1H-imidazole; hydrochloride. It has a decongestant property through mimicking the sympathetic influence on alpha-adrenergic receptors. It plays an important role in managing allergic conjunctiva, as it can reduce eye swelling and edema by acting on those receptors in the conjunctiva arterioles [3]. NPZ is an authorized drug in the United States (USP) [4] and British (BP) [5] pharmacopeias where its determination was carried out by HPLC methods. Moreover, the BP states four specified official impurities; one of them is impurity B (NPZ impurity B). On reviewing literature, NPZ has been determined as a single drug or in combination using several techniques, namely; spectrophotometry [6–14], HPLC [15–27], TLC [28] and capillary electrophoresis [29–36].

Pheniramine maleate (PHN) is chemically designed as (Z)-but-2-enedioic acid; N, N-dimethyl-3-phenyl-3-pyridin-2-ylpropan-1-amine. It is widely available in eye drops due to its antihistaminic and anticholinergic effect [37]. PHN is official in USP and BP whereas HPLC technique was reported for its assay. Two impurities were stated in BP for PHN, namely; A & B [5]. The literature survey revealed different techniques for its quantification either in single or in combined form, such as titrimetric [38], spectrophotometric [39, 40], HPLC [17–19, 27, 41–48] and capillary electrophoretic ones [36, 49].

**Fig. 1:**
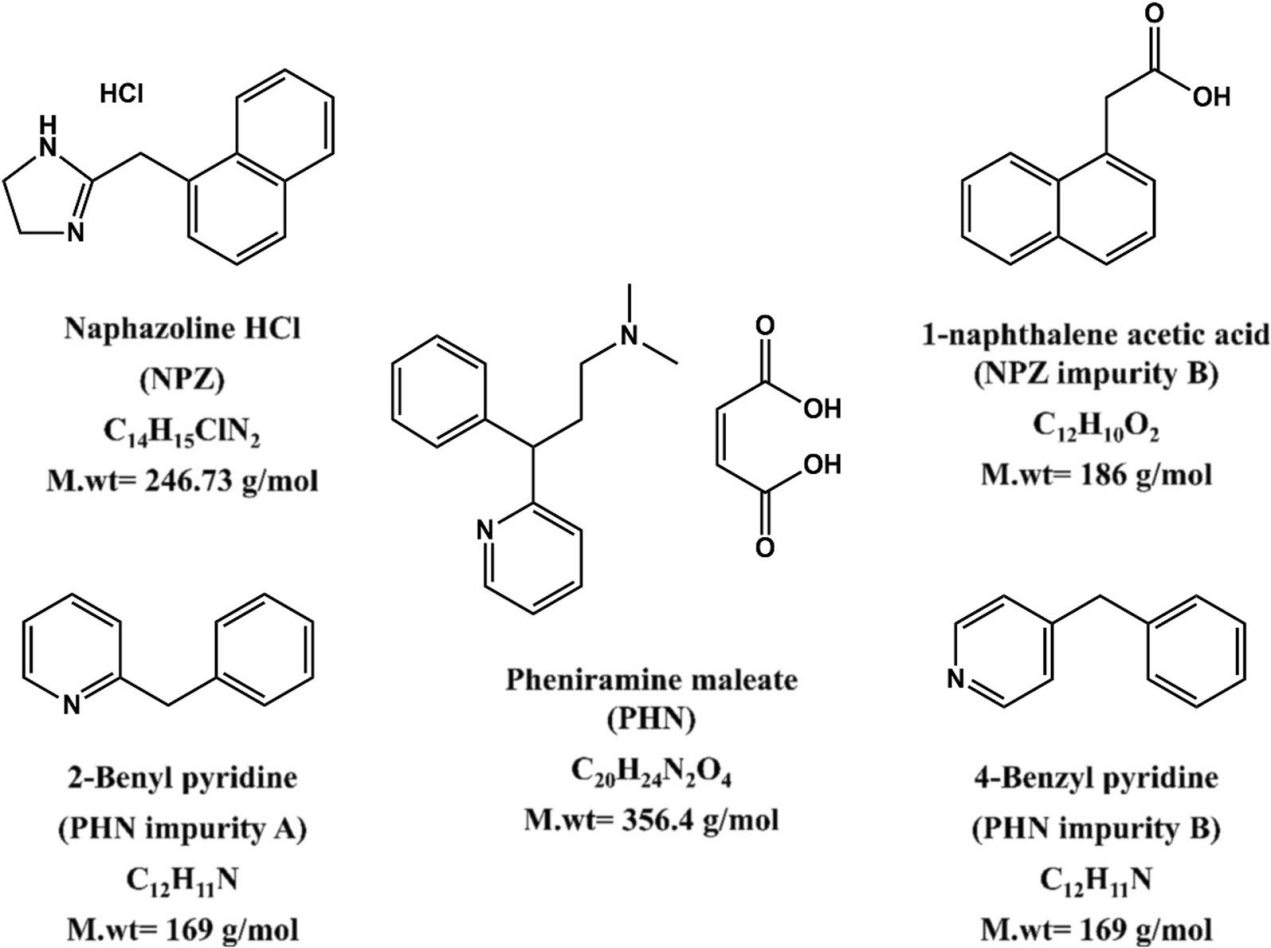
Chemical structures of the five cited components

NPZ and PHN are usually co-formulated together in optic dosage forms used for eye inflammation treatment. The literature survey revealed some methods for their simultaneous quantification, such as HPLC [17–19, 27] and one capillary electrophoresis [36]. One of the reported HPLC methods described their determination in presence of three selected impurities [19]. As a result, the aim of this work was to develop a first validated, as per ICH guidelines [50], TLC densitometric method for detection and quantification of NPZ, PHN, NPZ impurity B, PHN impurities; A and B, (Fig. 1). The proposed method was successfully applied for their simultaneous determination in a quinary mixture as well as in pharmaceutical eye drops. Furthermore, the organic solvents used in this work were assessed and compared to that used in the reported HPLC one [19] by the aid of environmental, health and safety (EHS) tool [51].

## Methods/Experimental

### Instruments

TLC system; a Camag Linomat autosampler (Muttenzl, Switzerland), a Camag micro syringe (100 µL), a Camag 35/N/30319 TLC scanner with win CATS software, UV lamp with a short wavelength at 260 nm (Desaga, Wiesloch, Germany) and TLC plates (10 × 20 cm) pre-coated with silica gel GF_254_ of 0.25 mm thickness (Merck, Darmstadt, Germany).

### Materials and chemicals

#### Pure standard

NPZ and PHN were kindly provided by Eva pharma pharmaceutical company, Cairo, Egypt. Their purities were assessed to be 100.12 ± 0.102% and 99.58 ± 0.124%, respectively [5]. The impurities (NPZ impurity B, PHN impurities; A & B) were purchased from the German company Alfa Aesar Company. Their certified potency values were found to be 99.00%, 100.30%, and 99.70%, respectively.

#### Pharmaceutical dosage form

Naphcon-A^®^ eye drops (Batch no.H13949-0615); manufactured by Alcon laboratories INC (Novartis Company), labeled to contain 0.25 & 3.0 mg mL^−1^ of NPZ and PHN, respectively, and has been purchased from Egyptian market.

#### Chemicals and reagents

Analytical-grade chemicals were used; methanol (Alpha, Egypt), ethyl acetate (Otsuka, Egypt), chloroform, acetone, 33.0% ammonia (El-Nasr, Egypt), hydrochloric acid (Sigma, Germany), 30.0% hydrogen peroxide solution (Adwic, Egypt) and sodium hydroxide pellets (Piochem, Egypt).

#### Solutions

##### Standard solutions

In 10-mL volumetric flasks, standard solutions of 20.0 mg mL^−1^, for NPZ, PHN and two PHN impurities, were separately prepared in methanol. For NPZ impurity B, 1.0 mg mL^−1^ standard solution was prepared.

##### Laboratory prepared mixtures

Different aliquots, from the five standard solutions, were transferred into separate 10-mL volumetric flasks to prepare laboratory prepared mixtures of various ratios. The volume of each flask was then completed to the mark using methanol.

### Procedures

#### Construction of the calibration curves

Aliquots equivalent to 2.0–50.0 mg of NPZ, 10.0–110.0 mg of PHN, 0.1–10.0 mg of NPZ impurity B and 2.0–50.0 mg of two PHN impurities (A & B) were transferred from their corresponding solutions into five sets of 10-mL volumetric flasks. Volumes were then completed to the mark with methanol. 10.0 µL from each solution was applied as a band with 3.0 mm length onto TLC plates (10 × 20 cm). A mobile phase of methanol: ethyl acetate: 33.0% ammonia (2.0: 8.0: 1.0, by volume) was used for elution over 8.5 cm distance. The elution time was around 6.0 min. After that, the plates were removed, air dried and scanned at 260.0 nm. Calibration curves, for the five cited drugs, representing the polynomial relationship between peak area and corresponding concentration were constructed and regression parameters were computed.

#### Assay of laboratory prepared mixtures

Different mixtures of NPZ, PHN and their official impurities were prepared as mentioned under solution section and mixed with different ratios. The prepared mixtures were then analyzed by the proposed method as mentioned above.

#### Forced degradation study

Stability of the two cited drugs were studied under different conditions, namely; acidic, alkaline, oxidative, photolytic and thermal ones. For acidic and alkaline hydrolyses, a mass of 10 mg of each drug was separately dissolved in least amounts of methanol then refluxing in 1 M HCl or methanolic solutions of 1 M NaOH at 100 °C for 2 h. For oxidative studies, 0.5 mL of 5% H_2_O_2_ aqueous solution was separately added to 10 mL of 1.0 mg mL^−1^ solutions. The two drugs solutions were then kept at room temperature for 24 h. For photolytic study, thin layers of each powdered drug was uniformly spread in two Petri dishes, and exposed to UV light at 254 nm for 10 h at a distance of 15 cm. Thermal degradation was assessed through sealing each powdered drug in glass ampoules and heating in a thermostatic oven at 100 °C for 7 h. Finally, samples were periodically withdrawn for observing the forced degradation process.

#### Pharmaceutical application

The content of 10 Naphcon-A^®^ eye drops were emptied. 20.0 mL aliquot was transferred into a 25-mL measuring flask. 3.0 mL methanol was added and the flask was then sonicated for 20.0 min. Volume was completed to the mark using methanol to obtain final concentration of 200.0 µg mL^−1^ NPZ and 2400.0 µg mL^−1^ PHN. 10.0 µLs from this solution were applied onto TLC plates. Finally, solutions were analyzed as mentioned before under construction of the calibration curves.

## Results and discussion

The importance of impurity detection and determination evokes the requirement for developing simple, economical, rapid and accurate analytical techniques which can be utilized easily in quality control laboratories wherein cost and time are essential. Owing to simplicity, cost effectiveness, time saving, no need for tedious sample preparation and high sensitivity, TLC densitometry could be considered as one of the best options for that purpose [2, 52, 53] Here, we present a novel TLC densitometric method for the simultaneous determination of NPZ, PHN and three related official impurities (NPZ impurity B, PHN impurities; A & B) in their quinary mixture. Moreover, EHS tool is applied for greenness evaluation of this method in comparison to our previously reported HPLC one [19].

### Development and optimization of TLC densitometric method

**Fig. 2:**
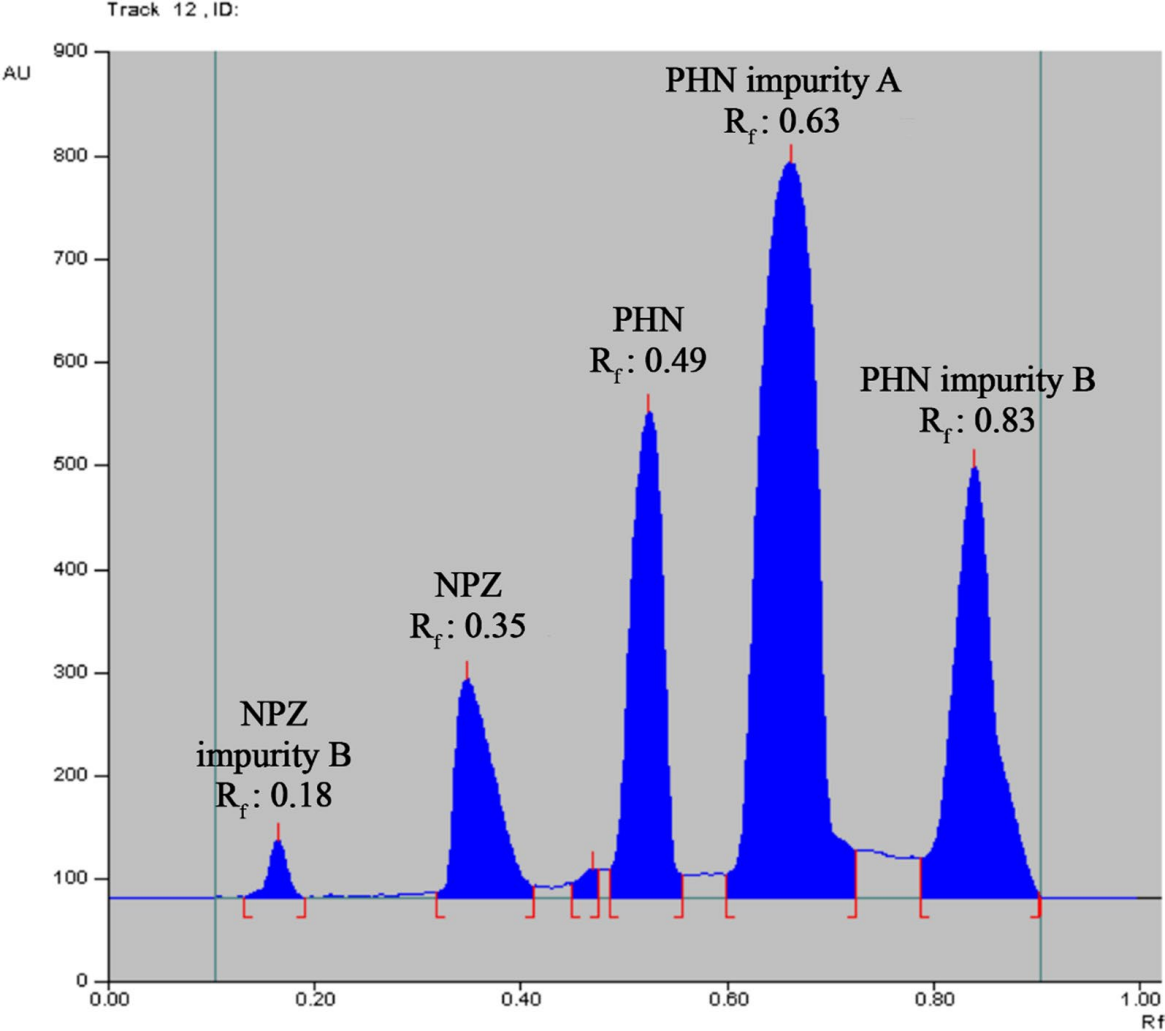
TLC chromatogram of NPZ (30.0 µg band ^−1^), PHN (90.0 µg band ^−1^) and three of their official impurities as NPZ impurity B (10.0 µg band ^−1^), PHN impurity A (40.0 µg band ^−1^) and PHN impurity B (40.0 µg band ^−1^) using a mobile phase of methanol: ethyl acetate: ammonia (2.0: 8.0: 1.0, by volume) and detection at 260.0 nm

Various mobile phases were tried to get optimum separation and resolution between the five cited components. Firstly, mixtures with different ratios of methanol and ethyl acetate have been tried, but the separation between the five cited components was not achieved. Thus, different solvents were added individually to the previous mixture for improving the separation between the studied components such as chloroform, acetone and ammonia. Table 1 summarizes the obtained resolution values during this optimization phase. It was noticed that addition of ammonia to the conventional mixture enhanced separation and resolution between the studied drugs. Finally, a mixture of methanol–ethyl acetate–ammonia (2.0: 8.0: 1.0, by volume) was chosen for optimum suitability parameters. Moreover, different wavelengths were tried for evaluating the densitometric measurement as 260.0 nm and 280.0 nm. 260.0 nm was the wavelength of choice as it gave the highest sensitivity with minimum noise for measuring the five cited components. Retardation factor (R_f_) values were sequentially at 0.18 ± 0.02, 0.35 ± 0.02, 0.49 ± 0.02, 0.63 ± 0.02 and 0.83 ± 0.02 for NPZ impurity B, NPZ, PHN, PHN impurity A and PHN impurity B (Fig. 2). Scanning profiles were obtained at 260.0 nm, and five calibration curves were then plotted.

**Table 1:** The obtained resolution values during mobile phase optimization

Experiment No.	Mobile phase composition	Rs1^a^	Rs2^a^	Rs3^a^	Rs4^a^
1	Methanol–ethyl acetate (3.5:6.5, v/v)	1.32	1.24	1.34	0.57
2	Methanol–ethyl acetate (3.0:7.0, v/v)	1.35	1.33	1.37	0.62
3	Methanol–ethyl acetate (2.5:7.5, v/v)	1.42	1.37	1.40	0.70
4	Methanol–ethyl acetate (2.0:8.0, v/v)	1.47	1.39	1.45	0.83
5	Methanol–ethyl acetate–chloroform (2.0:8.0:1.0, v/v/v)	1.49	1.51	1.13	0.75
6	Methanol–ethyl acetate–chloroform (2.0:8.0:0.8, v/v/v)	1.52	1.53	1.22	0.78
7	Methanol–ethyl acetate–chloroform (2.0:8.0:0.5, v/v/v)	1.53	1.54	1.27	0.89
8	Methanol–ethyl acetate–chloroform (2.0:8.0:0.2, v/v/v)	1.55	1.57	1.35	0.94
9	Methanol–ethyl acetate– acetone (2.0:8.0:1.0, v/v/v)	1.46	1.44	1.21	1.13
10	Methanol–ethyl acetate– acetone (2.0:8.0:0.8, v/v/v)	1.47	1.47	1.23	1.17
11	Methanol–ethyl acetate– acetone (2.0:8.0:0.5, v/v/v)	1.49	1.48	1.27	1.22
12	Methanol–ethyl acetate– acetone (2.0:8.0:0.2, v/v/v)	1.51	1.49	1.36	1.28

^a^Rs1, Rs2, Rs3 and Rs4 are the obtained resolutions between NPZ impurity B & NPZ, NPZ & PHN, PHN & PHN impurity A and PHN impurity A & PHN impurity B, respectively

### System suitability parameters

To evaluate the performance of the proposed TLC method, system suitability parameters were calculated manually [54]. The results of retardation, resolution, capacity and tailing factors for the five components were obtained in Table 2.

**Table 2:** Parameters required for system suitability tests of TLC densitometric method

Parameter	NPZ impurity B	NPZ	PHN	PHN impurity A	PHN impurity B
R_f_	0.18	0.35	0.49	0.63	0.83
Resolution (Rs)	NA	1.50	1.58	1.40	1.64
Tailing factor (T)	1.50	0.80	1.30	1.20	1.20
Retention factor (k')^a^	4.56	1.86	1.04	0.59	0.20
Selectivity factor (α)^b^	NA	2.45	1.79	1.76	2.95
Column efficiency (N)^c^	262.44	196.00	635.04	425.11	737.86
Height equivalent to theoretical plate (mm)	0.034	0.046	0.014	0.021	0.012

^a^Retention factor (k') = (1 − R_f_)/R_f_

^b^Calculation of α = k'2/k'1

^c^Column efficiency (N) = 16 (z/w)^2^, where z is the migration length of the spot, w is the spot width

### Method validation

Method’s validation was conducted in agreement to ICH guidelines [50].

#### Linearity and range

Polynomial relationships were established between the integrated peak area and the corresponding concentration in the ranges of 2.0  − 50.0 µg band ^−1^, 10.0–110.0 µg band ^−1^, 0.1  − 10.0 µg band ^−1^ and 2.0  − 50.0 µg band ^−1^ for NPZ, PHN, NPZ impurity B and the two PHN related impurities, respectively.

#### Accuracy

Accuracy was assessed by applying the previously mentioned procedures on pure samples with various concentrations within the defined ranges. Satisfactory results regarding recovery % were computed in Table 3.

**Table 3:** Regression parameters for determination of the studied drugs by the proposed TLC densitometric method

Parameter	NPZ impurity B	NPZ	PHN	PHN impurity A	PHN impurity B
Range	0.1–10.0 µg band^−1^	2.0–50.0 µg band^−1^	10.0–110.0 µg band^−1^	0.2–50.0 µg band^−1^	0.2–50.0 µg band^−1^
Slope	No. 1^a^ = − 172.85 No. 2^a^ = 3480.76	No. 1^a^ = − 13.91 No. 2^a^ = 1389.10	No. 1^a^ = − 1.82 No. 2^a^ = 584.57	No. 1^a^ = − 9.78 No. 2^a^ = 943.45	No. 1^a^ = − 11.64 No. 2^a^ = 962.46
Intercept	1343.80	3305.64	4730.10	14,708.36	11,166.09
SE of the slope	No. 1^a^ = 11.13 No. 2^a^ = 112.36	No. 1^a^ = 0.54 No. 2^a^ = 26.87	No. 1^a^ = 0.10 No. 2^a^ = 15.50	No. 1^a^ = 0.45 No. 2^a^ = 23.73	No. 1^a^ = 0.59 No. 2^a^ = 25.15
SE of the Intercept	182.40	249.72	492.06	232.59	202.55
Specificity ^b^ (mean ± SD)	99.11 ± 1.382	100.72 ± 0.221	100.25 ± 1.054	99.10 ± 1.152	98.89 ± 1.963
Accuracy	99.74	101.15	100.42	99.97	100.99
Repeatability (RSD)	1.28	1.47	1.29	0.74	1.03
Intermediate precision (RSD)	1.77	0.55	1.84	0.96	1.79
Robustness	0.98	0.78	0.84	1.07	1.45
LOD (µg band^−1^)	0.01	0.60	2.38	0.05	0.06
LOQ (µg band^−1^)	0.03	1.82	7.21	0.15	0.18
Correlation coefficient (r)	0.999	0.999	0.999	0.999	0.999

^a^Slope 1 and 2 are the coefficients of a polynomial regression, A = ax^2^ + bx + c, where A is the integrated peak area, x is the concentration of the drug (μg band^−1^), a and b are coefficients 1 and 2, respectively, and c is the intercept

^b^Average of determinations in seven laboratory-prepared mixtures

#### Precision

##### Repeatability

Three separate concentrations of NPZ (15.0, 25.0, 40.0 µg band^−1^), PHN (30.0, 50.0, 70.0 µg band^−1^), NPZ impurity B (3.0, 5.0, 8.0 µg band^−1^), PHN impurities; A & B (15.0, 25.0, 40.0 µg band^−1^) were analyzed intra-daily three times. Results were obtained preliminary to RSD calculation, Table 3.

##### Intermediate precision

Inter-daily analysis was also conducted for the formerly selected concentrations. Results are represented in Table 3.

#### Robustness

It was evaluated by studying the effect of deliberately changing the mobile phase composition; methanol (2.0 ± 0.2 mL), ethyl acetate (8.0 ± 0.2 mL) and ammonia (1.0 ± 0.1 mL). This study was conducted on three independent concentrations of NPZ (5.0, 20.0, 40.0 µg band^−1^), PHN (15.0, 40.0, 70.0 µg band^−1^), NPZ impurity B (1.0, 5.0, 8.0 µg band^−1^) and two PHN impurities (1.0, 20.0, 40.0 µg band^−1^ each). Satisfactory RSDs were obtained, Table 3.

#### Specificity

**Fig. 3:**
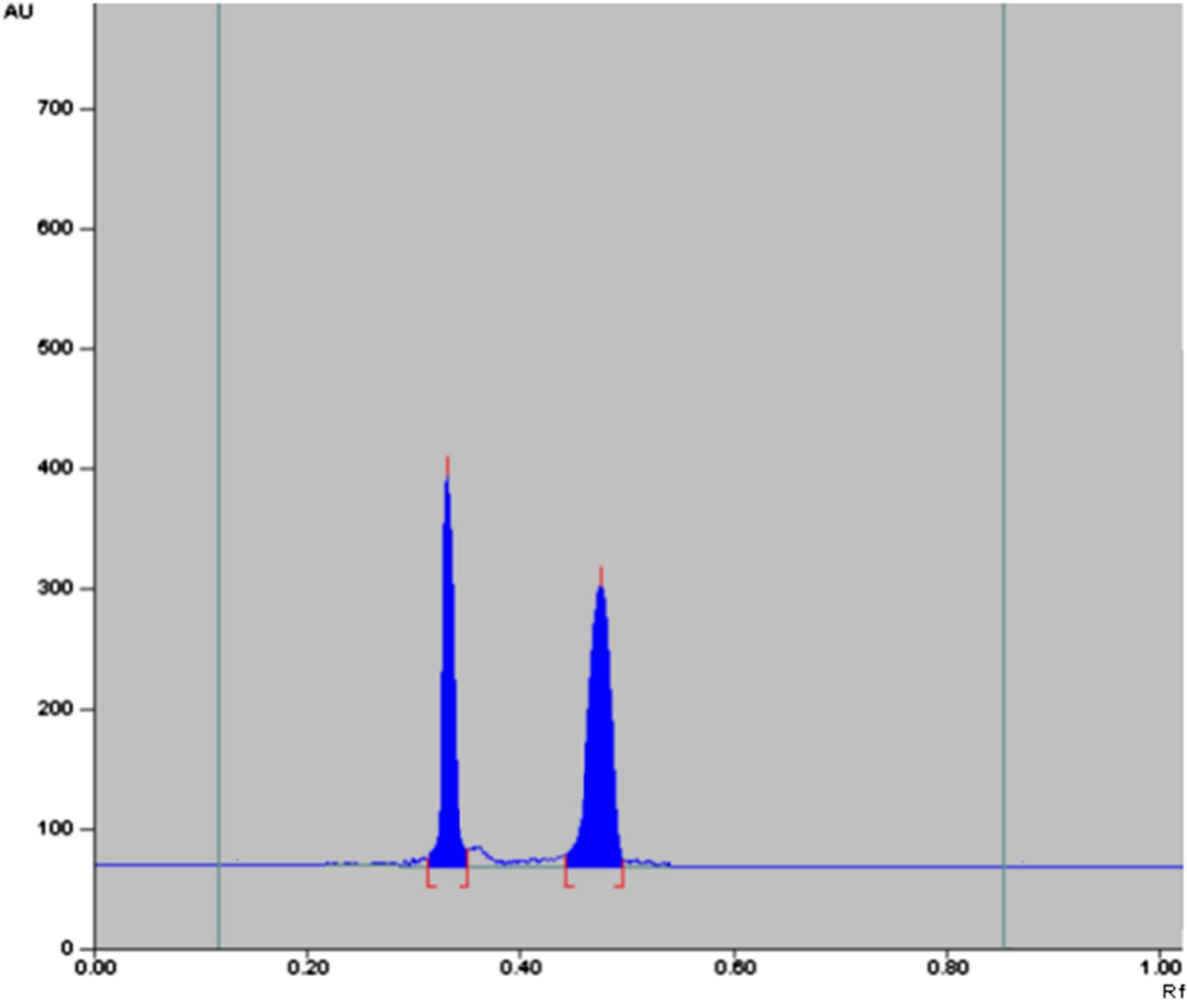
TLC chromatogram of 100 µg band^−1^ PHN after H_2_O_2_ treatment; R_f_ ≈ 0.49 for PHN & ≈ 0.32 for its oxidative degradation

It was assessed by analysis of different laboratory prepared mixtures containing various ratios of the five studied components. Table 3 shows good recovery percentages and RSDs for analyzing those mixtures. Furthermore, forced degradation study was conducted whereas the two drugs were subjected to different stress conditions: (1) Acidic and alkaline hydrolysis via refluxing with 1 M HCl and 1 M NaOH for 2 h, respectively, (2) Oxidative degradation through treatment of each solution with 5% H_2_O_2_ then keeping at room temperature for 24 h, (3) Photostability on powdered drugs placed in petri dishes, using 254 nm UV light for 10 h, and finally (4) Dry heat by putting each drug powder in 100 °C oven for 7 h. The degradation study was monitored by the proposed TLC method. NPZ was only liable to alkaline hydrolysis giving its impurity B where its spot (R_f_ ≈ 0.35) disappeared accompanied by appearance of a new one (R_f_ ≈ 0.18) corresponding to this specified impurity. This outcome is consistent with what previously reported [20]. PHN was stable towards all conditions except for oxidation where ≈ 30.0% was degraded upon H_2_O_2_ treatment (Fig. 3).

### Analysis of pharmaceutical eye drops

The two active pharmaceutical ingredients (NPZ and PHN) were simultaneously quantified in their combined dosage form. Excipients did not have an impact on the obtained TLC chromatograms. In addition, method’s validity was proved using standard addition technique, Table 4.

**Table 4:** Determination of NPZ, PHN in their dosage form and application of standard addition technique using the proposed TLC method

Naphcon-A^®^ eye drop	% found Mean^a^ ± SD	Standard addition technique		
		Taken	Added	Recovery %
NPZ	101.36 ± 1.51	10.0 µg band^−1^	5.0 µg band^−1^	101.30
			10.0 µg band^−1^	101.75
			20.0 µg band^−1^	99.99
Mean ± SD				101.01 ± 0.914
PHN	100.94 ± 1.73	20.0 µg band^−1^	10.0 µg band^−1^	100.95
			20.0 µg band^−1^	99.02
			40.0 µg band^−1^	100.84
Mean ± SD				100.27 ± 1.084

^a^Average determinations of four eye drop dosage form solution

### Statistical analysis

Statistical comparison between results of the suggested TLC method and that of official HPLC ones [5] were performed. The calculated values of student’s t-test and F-test indicated that there is no significant difference observed between those methods, Table 5.

**Table 5:** Statistical comparison between the results obtained by the proposed method and the official BP method

Parameter	TLC		Official BP method [5]	
	NPZ	PHN	NPZ	PHN
Mean of recoveries	101.15	100.42	99.63	99.71
SD	1.095	1.712	0.977	1.153
Variance	1.199	2.931	0.955	1.329
n	5	5	5	5
Student’s t-test	2.316 (2.306)^a^	0.778 (2.306)^a^	NA	NA
F-test	1.26 (6.39)^a^	2.21 (6.39)^a^	NA	NA

^a^These values represent the corresponding tabulated values of t and F at p = 0.05

### Greenness evaluation and methods comparison

In order to assess and compare this work with our previously reported HPLC one [19], EHS tool was applied. In this tool, nine categories representing safety, health and environmental hazards are utilized for organic solvents assessment whereas the lower the calculated score, the greener the solvent will be [51]. The calculated scores for methanol, ethyl acetate (used in this work) and acetonitrile (used in reported HPLC) revealed the dominance of the proposed method over our previously reported one in terms of environmental sustainability, Table 6. Finally, a comparative overview on those two methods along with a statistical F-test for their variances are shown in Table 7.

**Table 6:** EHS assessment of the solvents used in this work (ethyl acetate & methanol) as well as the reported one (acetonitrile)

Selected substance	Safety			Health			Environment			Total^a^
	Release potential	Fire/Explosion	Reaction/Decomposition	Acute toxicity	Irritation	Chronic toxicity	Persistency	Air Hazard	Water Hazard	
Acetonitrile	0.61	1.00	0.60	0.51	0.63	0.43	0.34	0.43	0.00	4.55
Ethyl acetate	0.62	1.00	0.00	0.28	0.63	0.17	0.03	0.17	0.00	2.89
Methanol	0.65	1.00	0.00	0.27	0.11	0.32	0.00	0.32	0.00	2.66

^a^Obtained by summation of nine main categories scores

**Table 7:** Comparative overview on reported HPLC and proposed TLC methods

Ref. No	LOD		Elution time	EHS score	F-test	
	NPZ	PHN			NPZ	PHN
[19]	1.29 µg mL^−1^	3.10 µg mL^−1^	≈ 30 min	4.55 (acetonitrile)	3.90 (6.39)^a^	2.22 (6.39)^a^
This work	0.60 µg band^−1^	2.38 µg band^−1^	≈ 6 min	2.89 (ethyl acetate) 2.66 (methanol)		

^a^This value represents the corresponding tabulated value of F at p = 0.05

## Conclusion

A novel simple TLC densitometric method was established for the simultaneous detection and quantification of NPZ, PHN as well as three of their official impurities (NPZ impurity B, PHN impurities; A & B). The proposed method was validated in agreement to ICH guidelines. NPZ and PHN were successfully determined in their combined eye drops. EHS tool was utilized for greenness assessment of the organic solvents used in this work as well as the previously reported HPLC one. The proposed TLC densitometric method provides simplicity, low cost, fast analysis and environmental sustainability compared to the reported one. In addition, the capacity of the method to detect low concentrations of NPZ and PHN official impurities highlights it as a promising one for impurity profiling of those drugs.

## Data Availability

All data generated or analysed during this study are included in this published article.

## Abbreviations

BP: British pharmacopeia
EHS: Environmental, Health and Safety tool
ICH: International Council for Harmonisation
NPZ: Naphazoline HCl
PHN: Pheniramine maleate
TLC: Thin Layer Chromatography
USP: United States Pharmacopeia
